# Problems with single platforms for CD34^+^ quantification: How aware are Brazilian hematologists and transplant specialists about them?

**DOI:** 10.1016/j.htct.2025.103836

**Published:** 2025-04-25

**Authors:** Daniel Mazza Matos

**Affiliations:** a Flow Cytometry Section, Cell Processing Center (CPC), Center of Hematology and Hemotherapy of Ceará (HEMOCE), Fortaleza, Ceará, Brazil; b Universidade de Fortaleza (UNIFOR), Fortaleza, Ceará, Brazil

*Dear Editor,*

I will start with a short anecdote. At the last XXVIII Congress of the Brazilian Society of Bone Marrow Transplantation, held in August 2024, I met with a longtime friend, an experienced hematologist with decades of expertise in various aspects of CD34 cell research, including quantification, collection, and cryopreservation. During our conversations, I mentioned that after reviewing the entire scientific program of the event, I did not find a single roundtable, symposium, or lecture devoted to the discussion of CD34 cells. In effect, during the entire Congress, there was only one presentation of a single study that addressed the quantification of CD34^+^ cells and its relationship with the success of leukapheresis. Considering the significance and centrality of CD34 cells in the execution of both autologous and allogeneic stem cell transplants, this observation was not just a curiosity − it was worrying, I told my friend. He agreed with me and said that, in fact, it would be advisable that at least a single symposium or roundtable should be devoted to the discussions on CD34 cells. “Yes,” I replied, “especially because, although very precise for CD34^+^ quantification, the modern single-platform templates that use microbeads for the enumeration of CD34^+^ cells are not free from problems.” Suddenly, he turned to me, his expression revealing a hint of surprise at what I had said, and asked, "What problems?"^1^ His response immediately made me think about how much Brazilian hematologists and transplant specialists are aware of the problems involving the quantification CD34^+^ cells.

Flow cytometry single-platform assays to enumerate CD34^+^ hematopoietic stem cells (CD34^+^ HSC) are the best methodology we currently have for the accurate and reliable determination of how many CD34^+^ HSC there are in each leukapheresis product intended for transplantation. In effect, over the past two decades, the single-platform technique became the ‘gold standard’ strategy for the quantification of CD34^+^ HSC for autologous and allogeneic hematopoietic stem cell transplantations (HSTC), surpassing the traditional International Society of Hematotherapy and Graft Engineering (ISHAGE)-based dual platform. As widely recognized, the principal advantage of the single-platform technique is its reduced variability as it excludes the need for white blood cell counts using automated hematology analysers [1,2].

Notwithstanding, single-platform assays are not without problems. In 2001, Bruno Brando et al. [3] described for the first time an uncanny phenomenon occurring with the single-platform method. The authors perceived that some of the microbeads present in the flow cytometry tube just vanished when phosphate-buffered saline (PBS)-diluted leukapheresis samples were vortexed before acquisition in the flow cytometer. As a result, the phenomenon generated artifactually high CD34^+^ HSC counts. They concluded that, when microbeads were resuspended in saline media, the vortex agitation almost invariably induced what they called the ‘vanishing counting bead’ (VCB) phenomenon. Nevertheless, although worrying, the problem of VCB is easy to solve: the addition of small amounts of protein (1% bovine serum albumin or 10% human pooled plasma) completely prevents the phenomenon. In order to avoid the VCB, the authors then advised that sample suspensions containing microbeads for single-platform analysis be resuspended in media containing protein supplements [3]. This guarantees precise CD34^+^ counting, preventing the realization of HSTC with a dose of CD34^+^ HSC that is below ideal.

After briefly explaining these points to my friend, I started to wonder whether Brazilian laboratories involved in the quantification of CD34^+^ HSC cells routinely supplement their samples with proteins and, furthermore, whether transplant physicians are aware of the possibility that the CD34^+^ HSC report they receive from general laboratories may contain inaccuracies due to the occurrence of VCB. So, preliminarily, my first intention with this letter is to share these concerns with other hematologists and transplant colleagues. But the issue is not so simple because, even if Brazilian laboratories are already adding proteins to avoid this problem, VCB- is like the Hydra from Greek mythology: it has many heads… or at least two heads.

In fact, we recently described a new problem with single-platform assays, a phenomenon we called ‘protein-resistant VCB’. In this case, VCB occurs even in protein-supplemented samples [4,5]. Although still awaiting the exclusion of local confounding variables that could be impacting this phenomenon, it appears that protein-resistant VCB is a very real, albeit rare, phenomenon, whose presence greatly increases the complexity of single-platform analyses. Therefore, it is important that Brazilian flow cytometry laboratories and transplant centers check whether they have encountered cases of classic (non-protein resistant) and of protein-resistant VCB and share their experience with the scientific community. Until the phenomenon is better defined and, more importantly, until we figure out how to eliminate it, we recommend that, in the presence of protein-resistant VCB, the dual-platform assay should be used for determining CD34^+^ HSC counts (Figure 1) [4,5].

**Figure 1:**
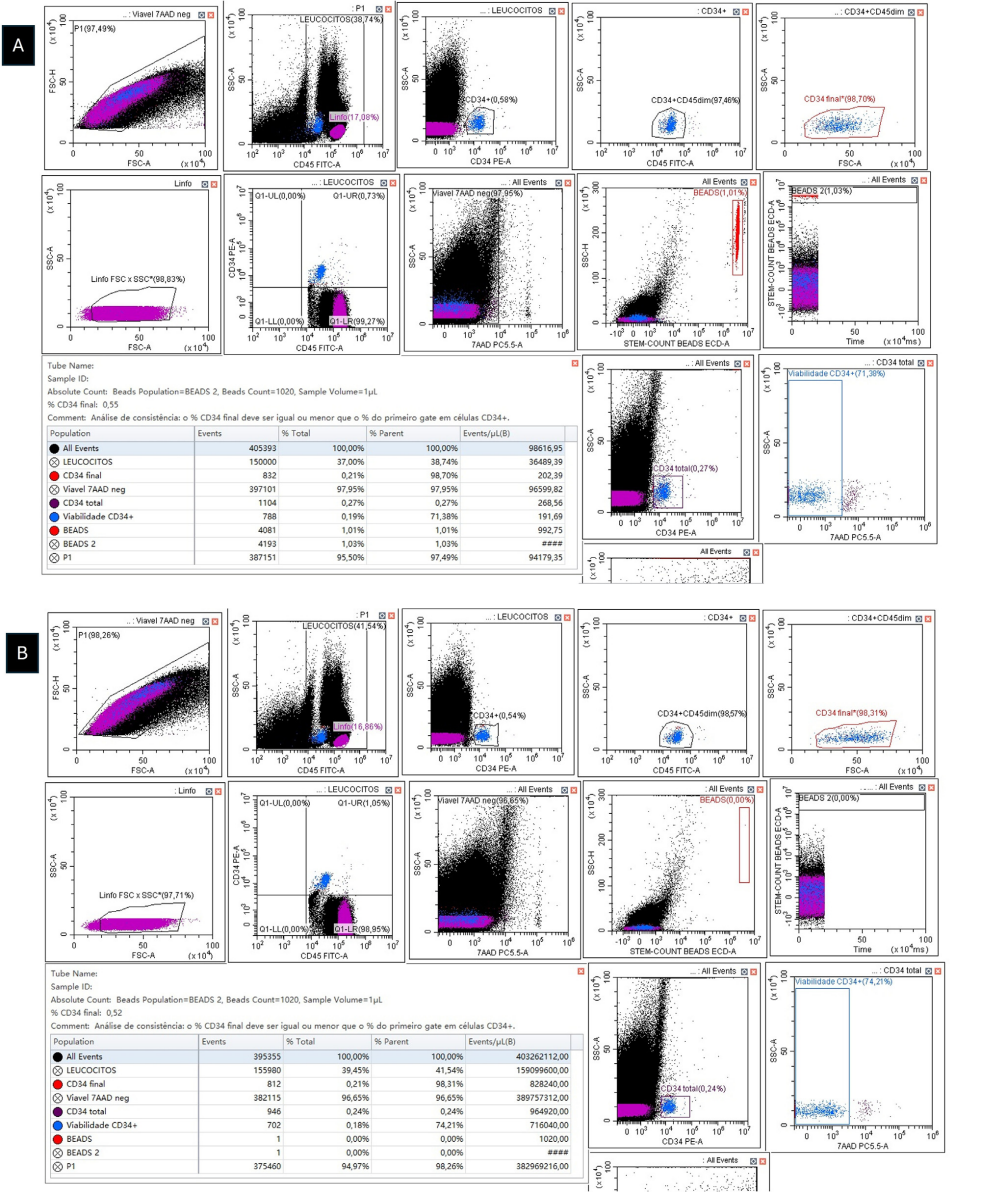
**A**) Enumeration of viable CD34^+^ cells with the single-platform ISHAGE protocol (Stem-Kit) on a DxFLEX flow cytometer (3-laser and 13-color detection) (Beckman Coulter). Leukapheresis sample incubated with CD34 PE, CD45 FITC, and 7-AAD plus 2% human albumin (dilution factor = 7). Viable CD34^+^ = 1.416 cells/mm^3^. **B**) Enumeration of viable CD34^+^ cells with the dual-platform ISHAGE protocol (Stem-Kit) on a DxFLEX flow cytometer (3-laser and 13-color detection) (Beckman Coulter). Leukapheresis sample incubated with CD34 PE, CD45 FITC, and 7-AAD plus 2% human albumin (white blood cell count = 143.700/mm^3^). Viable CD34^+^ = 748 cells/mm^3^. The dual-platform assay showed that the single-platform overestimated the viable CD34^+^ count, confirming the protein-resistant VCB phenomenon. Notice that a simple way to suspect the phenomenon is to use what we call ‘internal dual-platform’ derived from the single-platform template. In practice, what we do is to compare the value of viable CD34^+^ cells/mm^3^ of the single-platform method with the calculated value of viable CD34^+^ cells/mm^3^ of the ‘dual platform’ that is intrinsically present in single-platform studies. The formula is: **Internal dual-platform = (CD34^+^ events ÷ CD45^+^ events) x WBC (mm^3^)**. In this case, the internal dual-platform viable CD34^+^ cell count was (832 ÷ 150.000) x 143.700 = 797 cells/mm^3^. This result is quite consistent with that obtained in the dual-platform assay (for more details, see Matos DM.^5^).

Back to my friend. When I explained to him about the existence of VCB, he commented that he was unsure how many physicians in transplant centers and flow cytometry laboratories involved in CD34^+^ cell quantification in Brazil were aware of this problem concerning single-platform assays. I told him that I had no idea either. I hope, with this letter, that Brazilian hematologists and transplant specialists become aware about the need to substantially increase their attention when dealing with CD34^+^ HSC quantification platforms that use bead-based methods.

## Conflicts of interest

The author declares no conflicts of interest.

## Footnotes

1 If the readers are at this moment asking themselves the same question, then a careful reading of this “letter to the editor” is of utmost importance.
